# Utility of GDF‐15 as a diagnostic biomarker in gastric cancer: an investigation combining GEO, TCGA and meta‐analysis

**DOI:** 10.1002/2211-5463.12537

**Published:** 2018-11-28

**Authors:** Jie‐yu Liu, Xing‐xuan Dong, Jia‐nan Lu, Yue Zhang, Kai‐fan Liu, Ling‐feng Liu, Qing‐zhi E, Xiao‐jing Lu, Jie‐yun Yin, Yue‐ping Shen

**Affiliations:** ^1^ Jiangsu Key Laboratory of Preventive and Translational Medicine for Geriatric Diseases School of Public Health Medical College of Soochow University Suzhou China; ^2^ School of Basic Medicine Medical College of Soochow University Suzhou China

**Keywords:** diagnostic, gastric cancer, GDF‐15, meta‐analysis

## Abstract

It was recently suggested that growth differentiation factor‐15 (GDF‐15) is associated with gastric cancer (GC) carcinogenesis. However, the diagnostic potential of GDF‐15 for GC remains unclear. To address this issue, we obtained RNA sequencing and microarray data from the Gene Expression Omnibus (GEO) and The Cancer Genome Atlas (TCGA) databases, and searched PubMed, Google Scholar and Web of Science for relevant literature. We then used STATA to perform a meta‐analysis. In total, reports of 253 GC patients and 112 healthy controls who contributed peripheral blood samples were taken from the four literature sources, while information on 754 GC tumor and 263 gastric normal tissues was drawn from TCGA and seven GEO datasets. The expression level of *GDF‐15* mRNA was significantly higher in tumor tissues than in normal tissues, with a standard mean difference (SMD) of 0.79% and a 95% confidence interval (95% CI) of 0.63–0.95. Consistently, the GDF‐15 protein in blood was significantly increased in GC patients as compared to controls (SMD **= 3.74, 95% CI = 1.81–5.68). In addition, based on information from TCGA and GEO datasets, the expression level of *GDF‐15* mRNA may be of use for the diagnosis of GC, with a combined sensitivity, specificity and odds ratio of 0.69 (95% CI = 0.58–0.79), 0.90 (95% CI = 0.84–0.93) and 6.32 (95% CI = 4.22–9.49), respectively. The summary receiver operating characteristic curve demonstrated that the area under the curve was 0.90 (95% CI = 0.87–0.93). The results suggest higher levels of GDF‐15 may be associated with GC tumorigenesis and may have the potential to be a diagnostic biomarker of GC.

**Abbreviations**
GC: gastric cancer
GDF‐15: growth differentiation factor‐15
GEO: Gene Expression Omnibus
SMD: standard mean difference
SROC: summary receiver operating characteristic
TCGA: The Cancer Genome Atlas

Gastric Cancer (GC) is the third leading cause of cancer‐related death worldwide 1, 2, 3, 4. By 2015, the total number of patients diagnosed with GC in China was approximately 485 000 and was growing at a speed of 2.63% per year 5. According to Cancer Statistics in China in 2015, the incidence and mortality rates of GC were 679.1 and 498.0 per 100 000, respectively 5. Similar to other cancers, GC is a multi‐factorial disease, and several genetic and epigenetic factors are involved in its etiology. Environmental risk factors including smoking, *Helicobacter pylori* infection and obesity are also suggested to contribute to GC carcinogenesis 1, 6, 7.

Most GC has no obvious symptoms at the early stage 8; additionally, it is often mixed up with gastritis 9, gastric ulcer and gastric chronic disease symptoms 10. Therefore, the majority of GC cases are diagnosed in an advanced stage, with poor prognosis and limited treatment options 3. Endoscopic biopsy is the best way to find GC before clinical symptoms; however, few patients would like to undergo endoscopy due to potential offensive side effects, including aspiration, pneumonia, bleeding and perforation. Hence, it is indispensable to develop more acceptable, convenient and non‐invasive diagnostic methods.

Growth differentiation factor‐15 (GDF‐15), also known as macrophage inhibitory cytokine 1 (MIC1) 11, is a dimeric cytokine belonging to TGF‐β superfamily involved in the regulation of macrophage activation 12, 13. Under normal physiological conditions, it is highly expressed in the placenta and the prostate, but not common in other organs 13, 14, whereas in response to an unfavorable milieu, such as inflammation, oxidative stress, injury, ischemia, telomere erosion and oncogene activation, its production is potently upregulated in a broad range of tissues. For example, GDF‐15 is reported to be highly expressed in various malignant cancers, and is associated with the proliferation, metastasis and prognosis of colon cancer, ovarian cancer, oral squamous cell carcinoma, melanoma and prostate cancer 15, 16, 17, 18.

Recently, several studies revealed that the expression level of GDF‐15 is higher in GC patients, compared with healthy controls 9, 11, 19, 20, 21. Nevertheless, there are still inconsistent results 22, 23, 24. Besides, the sample size in these studies was relatively small and brought concerns about the robustness of the results. Therefore, we aimed to explore the expression pattern and diagnostic role of GDF‐15 in GC by utilizing public data and performing a meta‐analysis.

## Materials and methods

### Search strategy and inclusion criteria

Initially, GC‐related RNA‐sequencing data were searched in the National Center of Biotechnology Information (NCBI) Gene Expression Omnibus (GEO; http://www.ncbi.nlm.nih.gov/geo/) up to 1 July 2018. The search strategy was as follows: (stomach OR gastric) AND (cancer OR carcinoma OR tumor OR neoplas* OR malignan* OR adenocarcinoma). Afterwards, suitable literature was searched in PubMed, Google Scholar and Web of Science, using a combination of the following mesh words: ‘gastric’ AND ‘cancer’ AND ‘GDF‐15’.

**Figure 1:**
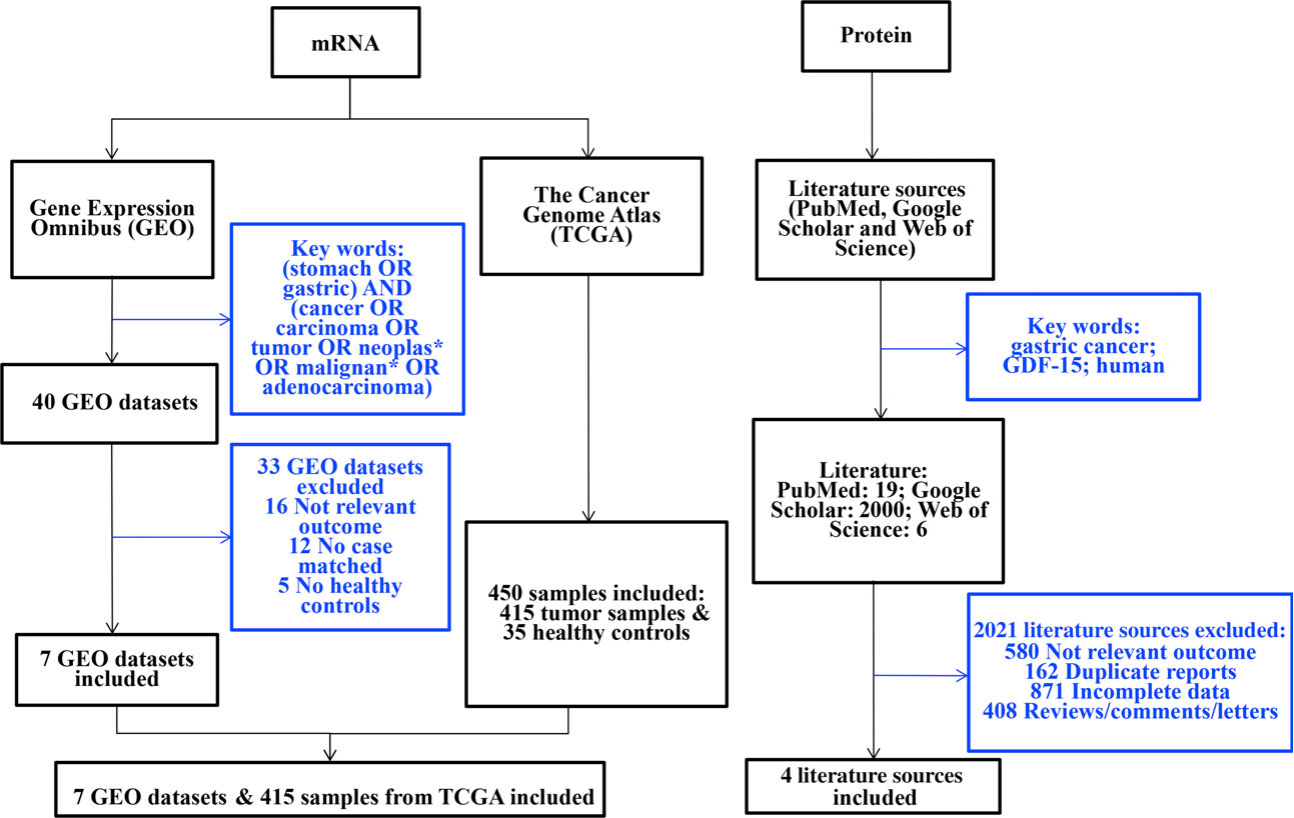
Flow diagram of literature selection.

The inclusion criteria of eligible data sets or literature were as follows. First, the study should evaluate the GDF‐15 mRNA or protein expression levels. Because the expression pattern of GDF‐15 in the body may vary with that in cell lines, which are cultured *in vitro*, we only collected the expression data from peripheral blood or tissues. Second, the expression of GDF‐15 was compared between GC patients and healthy controls, or between GC tumor and normal tissues. Third, the expression data of GDF‐15 and its mean and standard deviation should be available or calculable. Fourth, only human samples were included. Figure 1 shows the flow diagram of the literature selection.

According to the inclusion criteria, seven GEO datasets (GSE2685, GSE13911, GSE19826, GSE79973, GSE29272, GSE54129, GSE38932) and four suitable literature sources 11, 20, 25, 26 were finally included.

Finally, we extracted the mRNA expression data of *GDF‐15* of GC tumor and normal tissues from The Cancer Genome Atlas Stomach Adenocarcinoma (TCGA‐STAD) dataset.

### Statistical analysis

Initially, the expression levels of GDF‐15 mRNA or protein were extracted from datasets or articles; the mean and standard deviation were calculated. Later, a meta‐analysis was performed to get pooled standard mean difference (SMD) with 95% confidence interval (95% CI), which indicated the expression differences. Statistical heterogeneity was tested by using Cochran's *Q* statistic and *I* ^2^ tests, and *P *< 0.05 and *I* ^2^ > 50% were considered to be statistically heterogeneous 27. Where this was so, a random‐effects model was conducted for combination. Otherwise, a fixed‐effects model was used. Additionally, funnel plots and Begg's test were used to check the potential publication bias. A one‐way sensitivity analysis (one study excluded at the time) was also performed. Finally, as the original data of protein expression from articles were not available, we only included *GDF‐15* mRNA expression data from TCGA and GEO datasets, and then conducted diagnostic odds ratio analysis and assessed the diagnostic possibility of GDF‐15 for GC patients. Another approach, meta‐analysis with summary receiver operating characteristic (SROC), was further carried out to verify the expression level of *GDF‐15* mRNA in GC.

## Results

As shown in Table 1, 754 tumor and 263 normal tissues were derived from seven GEO (GSE2685, GSE13911, GSE19826, GSE79973, GSE29272, GSE54129, GSE38932) and TCGA datasets. At the same time, the protein expression levels of GDF‐15 in peripheral blood of 253 GC patients and 112 controls were obtained from four literature sources.

**Table 1:** Characteristics of GDF‐15 expression profiling datasets included in the current meta‐analysis

Dataset	Country	Sample type^a^	Platform	Tested substance	Tumor tissue/Case			Normal tissue/Control		
					No.	Mean	SD	No.	Mean	SD
GSE2685	Japan	Tissues	GPL80	mRNA	22	373.52	225.35	8	227.38	133.58
GSE13911	Italy	Tissues	GPL570	mRNA	38	3645.12	3353.66	31	785.59	448.62
GSE19826	China	Tissues	GPL570	mRNA	12	1426.26	815.20	12	525.28	156.66
GSE79973	China	Tissues	GPL570	mRNA	10	9.86	1.30	10	9.19	0.60
GSE29272	USA	Tissues	GPL96	mRNA	134	7.50	1.53	134	6.68	0.89
GSE54129	China	Tissues	GPL570	mRNA	111	7.55	1.37	21	6.48	0.43
GSE38932	Argentina	Tissues	GPL5936	mRNA	12	−0.06	0.36	12	−0.28	0.24
TCGA		Tissues		mRNA	415	1943.80	2137.17	35	277.69	267.26
M. Blanco‐Calvo 11	Spain	Peripheral blood		Protein	52	453.36	357.13	23	212.22	84.79
T. Ishige 25	Japan	Peripheral blood		Protein	62	1159.00	579.00	22	383.00	110.00
R. J. E. Skipworth 26	UK	Peripheral blood		Protein	103	1592.00	2083.67	35	377.00	911.25
L. Lu 20	China	Peripheral blood		Protein	36	14.28	1.03	32	1.05	0.21

SD, standard deviation.

a *GDF‐15* mRNA expression was compared between tumor tissues and normal tissues from gastric cancer patients, while GDF‐15 protein levels in peripheral blood were compared between gastric cancer patients and healthy controls.

**Figure 2:**
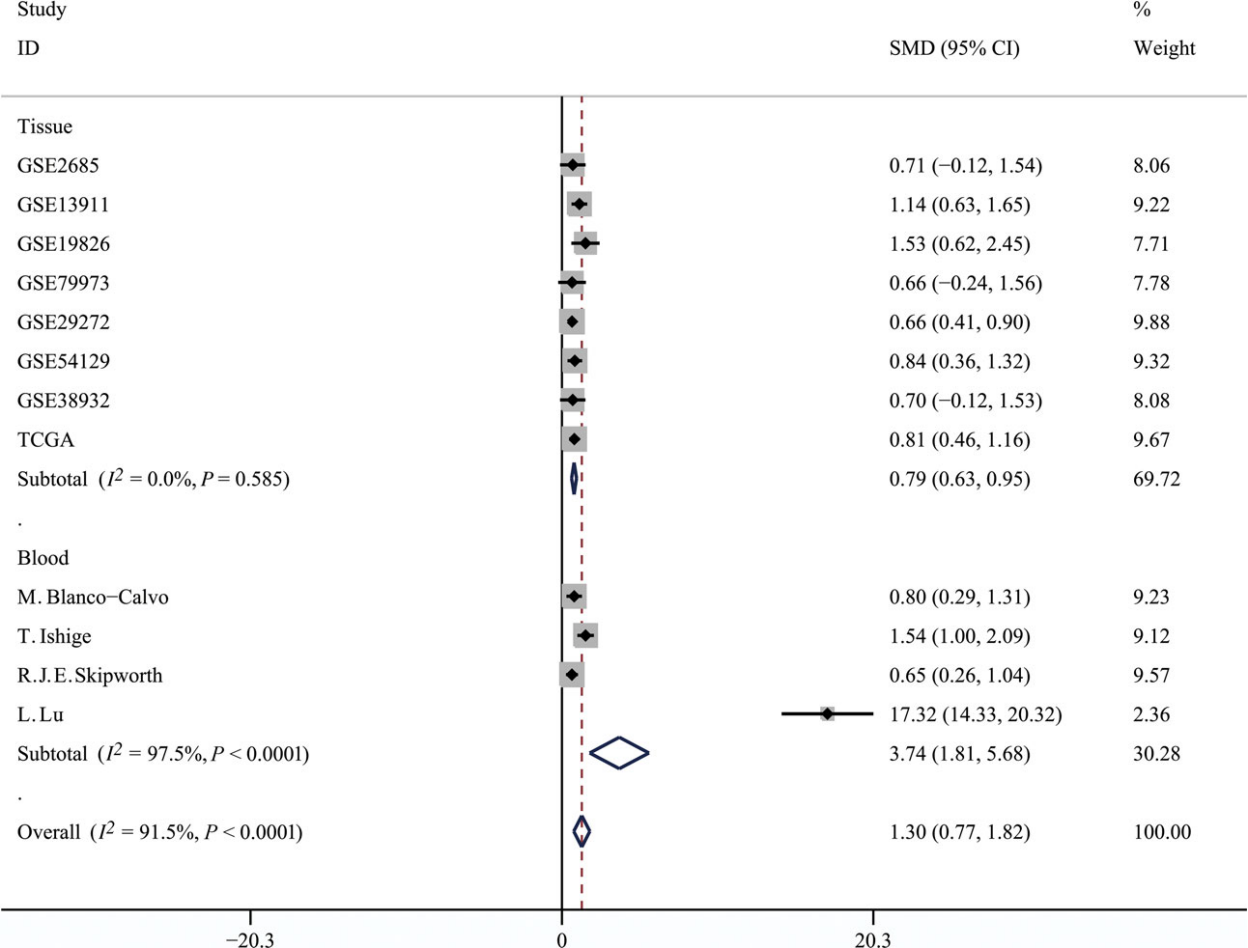
Forest plot showing SMD of GDF‐15 expression between tumor and normal tissues of gastric cancer patients, and between blood of gastric cancer patients and normal controls. Fixed‐effects model was used for tissue group, and random‐effects model was used for blood group.

**Figure 3:**
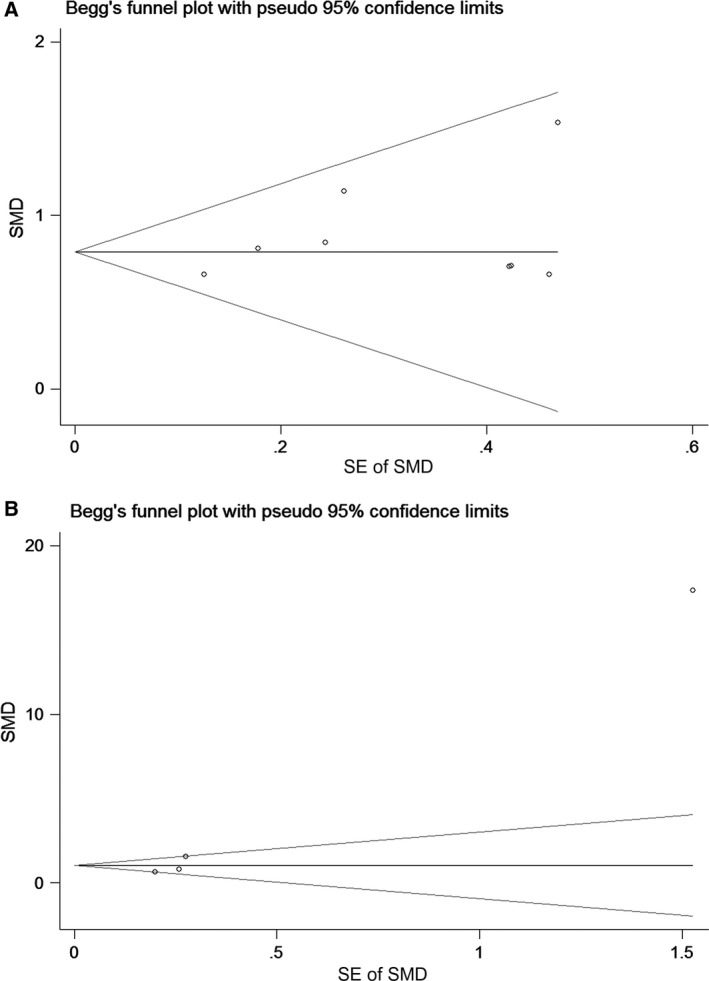
Begg's funnel plot for the assessment of potential publication bias in the tissue group (A) and the blood group (B).

Figure 2 shows that *GDF‐15* mRNA expression was significantly increased in GC tumor tissues compared with normal gastric tissues, with a SMD of 0.79 (95% CI = 0.63–0.95). Also, the expression levels of GDF‐15 protein were obviously higher in GC patients than in healthy controls (SMD* *= 3.74, 95% CI = 1.81–5.68). Besides, no publication bias existed for the tissue group (*P* for Begg's test = 0.536) and for the blood group (*P* for Begg's test = 0.089), as shown in Fig. 3. Additionally, the result remained stable according to the sensitivity analysis (Fig. S1).

**Figure 4:**
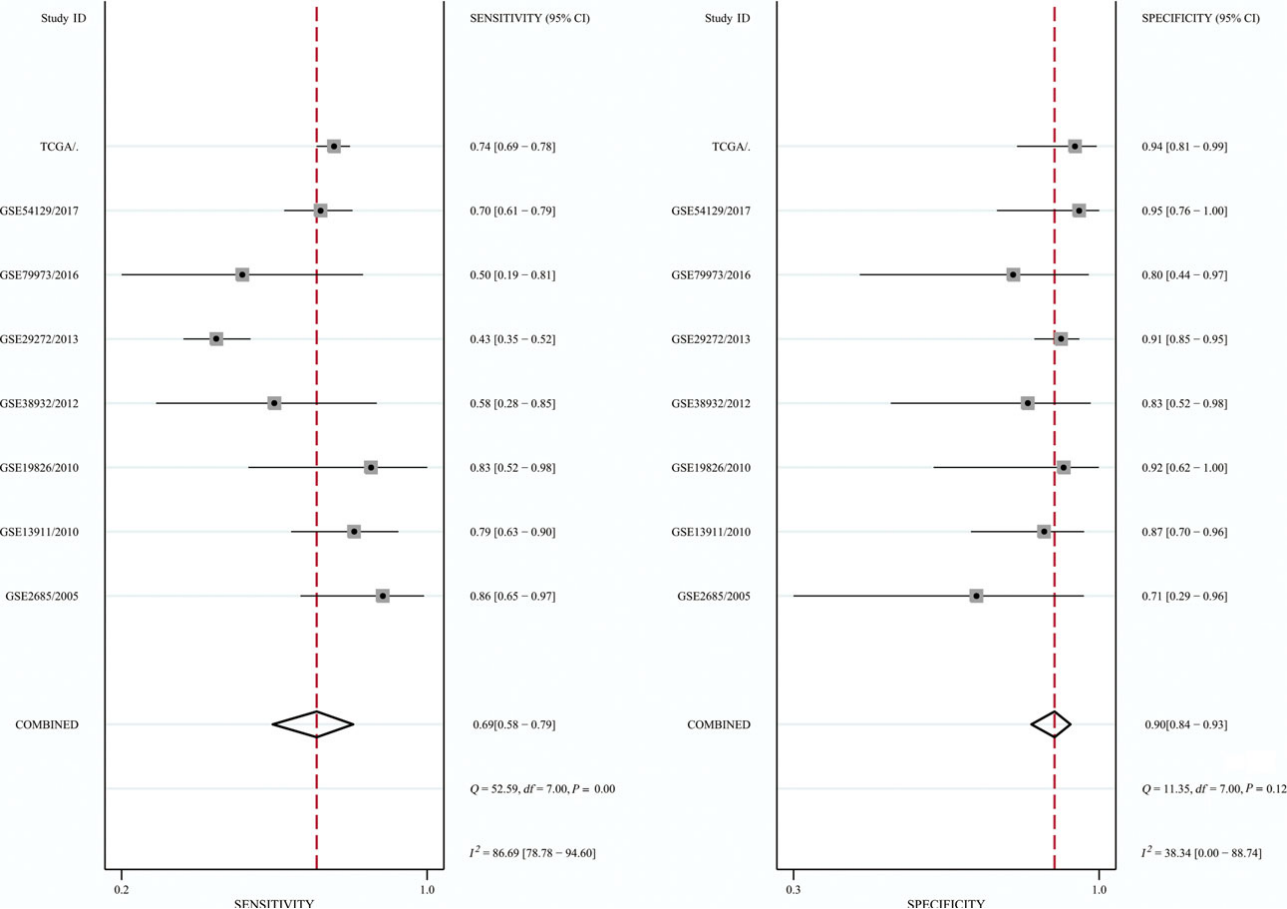
Diagnostic analysis of tissue *GDF‐15* mRNA in gastric cancer.

**Figure 5:**
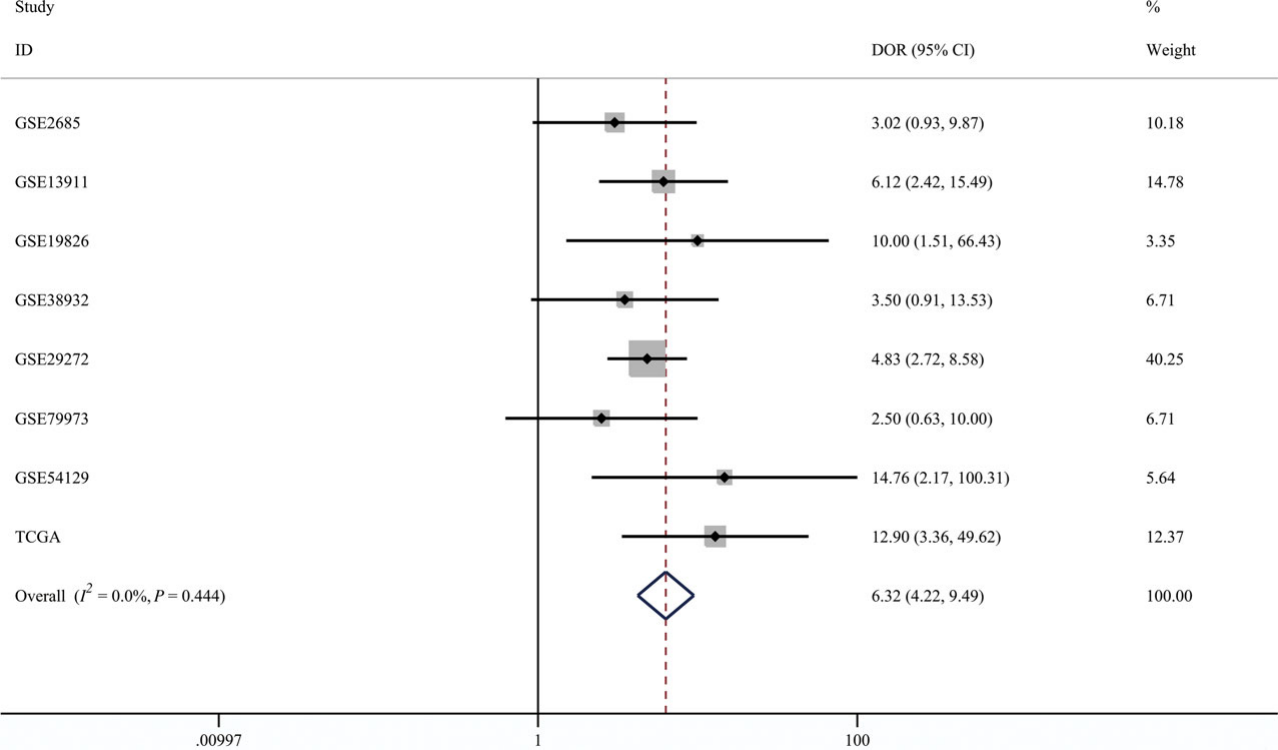
Forest plot of the diagnostic value of tissue *GDF‐15* mRNA in gastric cancer.

**Figure 6:**
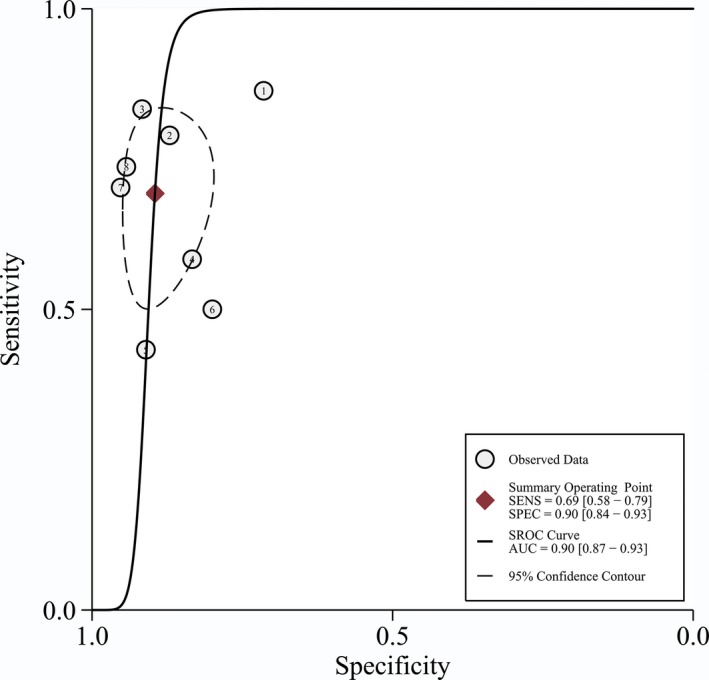
Summary receiver operating characteristic curve of tissue *GDF‐15* mRNA in gastric cancer. AUC, area under curve.

Turning to the diagnostic analysis, 754 tumor and 263 normal tissues derived from the TCGA and GEO datasets were included. Figures 4 and 5 illustrate that the combined sensitivity, specificity, and odds ratio are 0.69 (95% CI = 0.58–0.79), 0.90 (95% CI = 0.84–0.93) and 6.32 (95% CI = 4.22–9.49), respectively. The SROC curve represented in Fig. 6 demonstrated that the area under the curve was 0.90 (95% CI = 0.87–0.93).

## Discussion

In the present study, it was found that *GDF‐15* mRNA was significantly increased in GC tumor tissues compared with normal gastric tissues, while GDF‐15 protein was over‐expressed in the blood of GC patients compared with healthy controls. The additional diagnostic meta‐analysis demonstrated that GDF‐15 had a potential diagnostic value for GC. In our results, GDF‐15 is highly expressed in GC and the area under the SROC curve was 0.90, which indicated a diagnostic value of GDF‐15 in GC tumors compared with non‐cancerous control. Hence, GDF‐15 may be considered as an early diagnostic biomarker and even a candidate therapeutic target for GC.

In line with our study, other researchers reported that GDF‐15 was over‐expressed in gastric cancer cell lines 9, 19. In addition, GDF‐15 was found to be correlated with progressive pathological parameters in GC 9. The underlying mechanisms of GDF‐15 in GC are not known in detail. Recent study has indicated that the circulating GDF‐15 level correlates weakly with systemic inflammation in advanced gastric cancer and may also contribute to fibroblast activation as well as TGF‐β 28. It was also uncovered that the stimulation by GDF‐15 of NIH3T3 fibroblasts could enhance proliferation and up‐regulate expression of extracellular matrix genes, which were involved in malignant progression 9. What is more, previous studies implied that GDF‐15 could stimulate the urokinase‐type plasminogen activator activation system 19 and induce ErbB2 transactivation 29, subsequently enhancing invasiveness of GC cells and eventually contributing to tumorigenesis.

It should be noted that this analysis has some limitations. The results obtained in different study datasets may vary depending on variant conditions. GDF‐15 levels might be influenced by many factors, including age, gender, smoking status, diabetes mellitus and so on. Depending on the information we extracted, we were unable to exclude the influence of the above‐mentioned variables. Besides, the present study is quite preliminary, and a study determining the pathophysiology of this relationship is urgently warranted.

## Conclusions

In conclusion, the present study suggests that a high level of GDF‐15 is associated with GC. In addition, GDF‐15 has the potential to serve as a biomarker for GC diagnosis. Further studies exploring the role of GDF‐15 in GC carcinogenesis are urgently needed.

## Author contributions

YP‐S and JY‐Y contributed to the design and conception of the study. The others have contributed to writing and revision of the paper. JY‐L and XX‐D: acquisition of data, analysis and interpretation of data, and manuscript drafting. KF‐L, YZ and JN‐L: acquisition of data, and analysis and interpretation of data. XJ‐L and QZ‐E: analysis and interpretation of data, and critical revision of the manuscript for intellectual content.

## Conflict of interest

The authors declare no conflict of interest.

## Supporting information


**Fig. S1.** Sensitivity analysis of the value of GDF‐15 in the diagnosis of GC.
