# Supplementary figures and images for: Utility of GDF‐15 as a diagnostic biomarker in gastric cancer: an investigation combining GEO, TCGA and meta‐analysis
Source: FEBS Open Bio. 2018 Nov 28;9(1):35–42. doi: 10.1002/2211-5463.12537 (PMC6325603; doi:10.1002/2211-5463.12537)

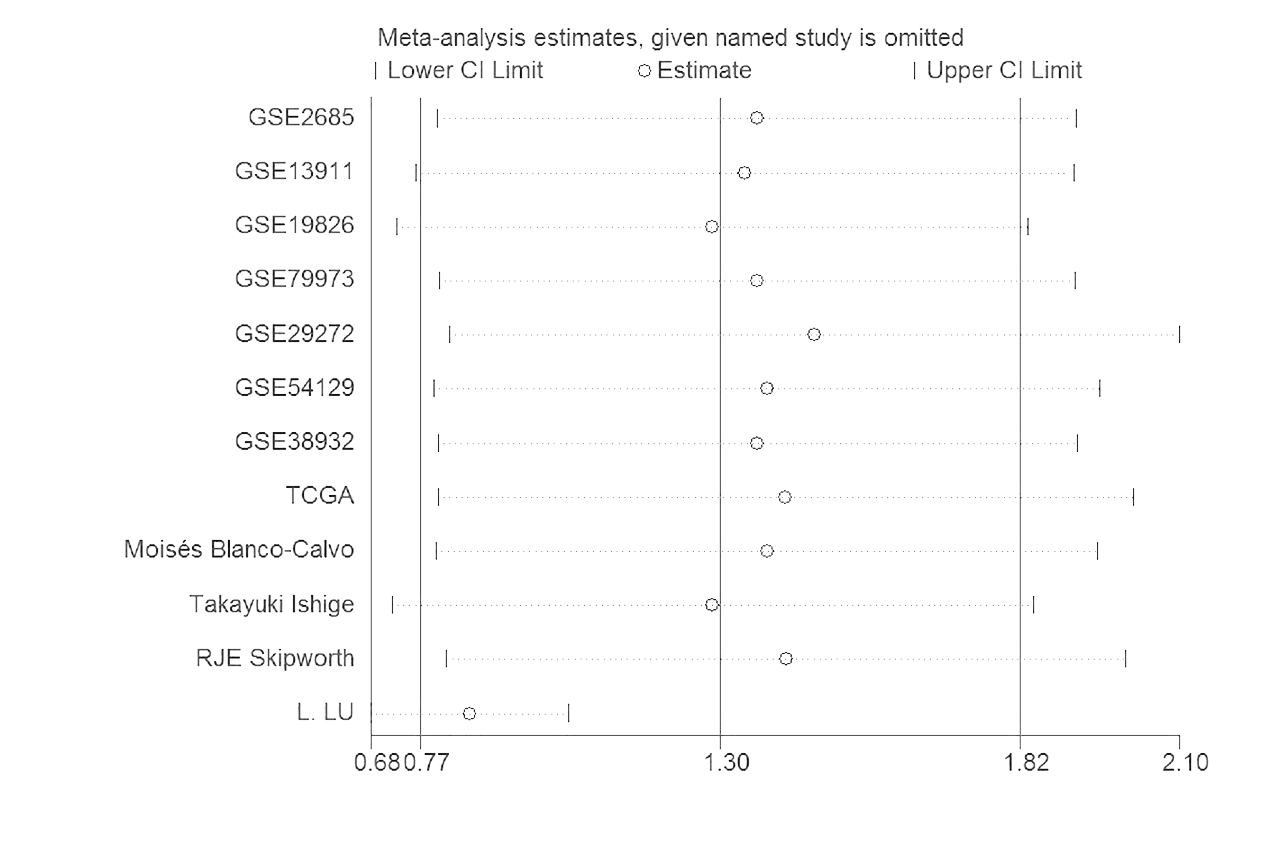
**Supplementary Figure 1.**Sensitivity analysis of the value of GDF-15 on the diagnosis of GC.

Supplement: Supplementary file 1 — Fig. S1. Sensitivity analysis of the value of GDF‐15 in the diagnosis of GC. [file FEB4-9-35-s001.docx]
